# Myelination, axonal loss and Schwann cell characteristics in axonal polyneuropathy compared to controls

**DOI:** 10.1371/journal.pone.0259654

**Published:** 2021-11-04

**Authors:** Eva Placheta-Györi, Lea Maria Brandstetter, Jakob Zemann-Schälss, Sonja Wolf, Christine Radtke

**Affiliations:** Department of Plastic and Reconstructive Surgery, Medical University of Vienna, Vienna, Austria; BG Trauma Center Ludwigshafen, GERMANY

## Abstract

**Introduction:**

Polyneuropathy is a debilitating condition characterized by distal sensory and motor deficits. Schwann cell dysfunction and axonal loss are integral factors in pathophysiology and disease progression of polyneuropathy.

**Aims:**

The aim of this study was the assessment of Schwann cell characteristics, nerve fibers and myelination parameters in polyneuropathy patients compared to controls.

**Methods:**

Nerve tissue was obtained from polyneuropathy patients (n = 10) undergoing diagnostic sural nerve biopsies. Biopsies of healthy peripheral nerves (n = 5) were harvested during elective sural nerve grafting for chronic peripheral nerve lesions. Exclusion criteria for the healthy control group were recent neurological trauma, diabetes, neurological and cardiovascular disease, as well as active malignancies and cytotoxic medication within the last 12 months. The over-all architecture of nerve sections and myelination parameters were histomorphometrically analyzed. Immunofluorescent imaging was used to evaluate Schwann cell phenotypes, senescence markers and myelination parameters.

**Results:**

Histomorphometric analysis of nerve biopsies showed significant axonal loss in polyneuropathy patients compared to controls, which was in accordance with the neuropathological findings. Immunofluorescent staining of Schwann cells and myelin basic protein indicated a significant impairment of myelination and lower Schwann cell counts compared to controls. Phenotypic alterations and increased numbers of non-myelinating p75-positive Schwann cells were found in polyneuropathy patients.

**Discussion:**

This study provided quantitative data of axonal loss, reduced myelination and Schwann cell dysfunction of polyneuropathy patients compared to neurologically healthy controls. Phenotypic alterations of Schwann cells were similar to those seen after peripheral nerve injury, highlighting the clinical relevance of Schwann cell dysfunction.

## Introduction

Polyneuropathy affects 2–4% of the general population. The prevalence increases to 8% in the population aged 55 years or older [1]. While the clinical presentation is heterogenous, distal symmetric polyneuropathy is the most common subtype [2]. Risk profiles and etiological factors differ globally with over 100 causes of polyneuropathy identified [3]. Diabetes is the most common risk factor with rising incidence, which accounts for 30–40% of polyneuropathy cases [4]. Idiopathic polyneuropathy affects approximately 20–30% of patients [3]. Diagnostic guidelines have been established to standardize diagnostic procedures and scientific reporting [5]. Algorithms include clinical and electrodiagnostic testing, serological markers, and skin and/or peripheral nerve biopsies [6, 7]. Sural nerve biopsy is recommended to detect demyelination and axonal damage, and to differentiate inflammatory disorders (e.g. vasculitis, sarcoidosis, CIDP), infectious diseases (e.g. leprosy), or amyloidosis [1, 8].

Although the etiologies of polyneuropathy are heterogenous, common pathophysiological pathways leading to neural damage have been described. Vascular and neurochemical mechanisms are linked to the development of diabetic polyneuropathy, which is the most common subtype in western countries [9]. Neurochemical changes affect different cells of the peripheral neural system such as Schwann cells, spinal cord oligodendrocytes and dorsal root neurons [9]. These changes include alterations in cellular metabolism, signal transduction and neurotrophic transport. Changes associated with diabetic polyneuropathy led to inadequate peripheral nerve regeneration, which was described as an important factor of disease progression. After injury, regeneration and sprouting does not only occur in the injured distal axons, but also in the dermis and close to the neurons of the dorsal root ganglia, contributing to the generation of pain sensation [10]. Overall, peripheral nerve regeneration is decreased in polyneuropathy, which was demonstrated clinically and in experimental models. Decreased levels of neurotrophic factors and their receptors, alterations in cellular signaling pathways, and altered expression of cell adhesion molecules have been described as contributing factors [10]. The critical role of Schwann cells in peripheral nerve regeneration has been thoroughly investigated [11, 12] and many of these findings translate to the development of polyneuropathy, as interactions between Schwann cells, axons and microvessels contribute to the disease [13]. Although much of the previous literature has been focused on axonopathy and vascular damage, the integral role of Schwann cells is gaining increasing evidence [13, 14]. Axons and Schwann cells can be independently affected and develop axonopathy and Schwann cell dysfunction, while their cellular and paracrine interactions may also be disrupted [13–15]. Experimental studies suggested that hypoxia, inflammation and immunological factors contribute to Schwann cell dysfunction in polyneuropathy, leading to loss of trophic support and ultimately to Schwann cell apoptosis [13, 16–19]. To date, there are only few reports on Schwann cell function in human polyneuropathy, however, first descriptions of morphological changes in Schwann cells of polyneuropathy patients date back to 1979 [20]. Experimental polyneuropathy models provided increasing evidence on Schwann cell phenotype changes and molecular alterations [13, 21]. The aim of this study was to characterize Schwann cell function and phenotypes in polyneuropathy patients compared to neurologically healthy adults, thereby investigating how experimental reports of Schwann cell dysfunction and axonopathy translate to clinical findings.

## Methods

### Patient characteristics

Patients suffering from polyneuropathy who underwent diagnostic sural nerve biopsies (n = 10) and neurologically healthy controls (n = 5), who underwent reconstructive surgeries that required selective denervations, were included in this study from June 2017 to February 2021. The local ethics review board granted approval of the study (protocol number: 2219/2016) and all patients gave their informed consent.

Inclusion criteria for the polyneuropathy group (Table 1) were adult patients with electroneurographically confirmed sensomotoric polyneuropathy, no recent peripheral nerve injuries in the last 12 months, no pregnancy, and no active malignancies or cytotoxic treatments.

**Table 1:** Patient characteristics. 10 Patients with polyneuropathy undergoing diagnostic sural nerve biopsy were included in this study.

***Patient***	***Sex***	***Age at Biopsy***	***Duration of symptoms***	***Suspected etiology***	***Neuropathological report***	***Diagnosis***
*1*	male	56 years	2 months	idiopathic	advanced, chronic axonal neuropathy	distal, symmetric axonal PNP
*2*	female	51 years	unknown	SLE-associated	advanced axonal neuropathy, CD3- and CD8 positive T-cells	SLE-associated chronic, axonal PNP
*3*	female	68 years	3 months	idiopathic	advanced, chronic axonal neuropathy, endoneural microangiopathy	chronic, symmetric axonal PNP
*4*	male	54 years	11 months	idiopathic	subacute and chronic axonal neuropathy, possibly metabolic-toxic	axonal, sensomotoric PNP
*5*	male	27 years	25 months	diabetic	advanced, chronic axonal neuropathy	chronic, axonal PNP
*6*	male	49 years	unknown	idiopathic	advanced, chronic axonal neuropathy	distal, symmetric PNP, small-fiber neuropathy
*7*	male	59 years	unknown	idiopathic	axonal neuropathy with de- and remyelination signs	chronic, axonal PNP
*8*	female	62 years	1 month	vasculitis-associated	pronounced, acute axonal neuropathy with perivascular lymphocytes	vasculitis-associated PNP
*9*	male	76 years	>30 years	CIPD	severe, chronic axonal neuropathy	chronic, axonal PNP
*10*	male	68 years	1 month	paraprotein-associated PNP	severe, chronic axonal neuropathy, changes compatible with toxic or paraprotein-associated PNP	paraprotein-associated, chronic, axonal PNP

PNP: Polyneuropathy; CIDP: chronic inflammatory demyelinating polyradiculoneuropathy; SLE: systemic lupus erythematosus.

Sural nerve biopsies were routinely performed in standard operative technique [22] with exposure of the sural nerve dorsal to the lateral malleolus. Routine neuropathological analysis of sural nerve tissue was performed and all nerve tissue obtained for this study was harvested without causing additional donor nerve deficits in the affected patients.

3 female and 7 male polyneuropathy patients were included in this study. The mean age in the polyneuropathy group was 57.3 years (SD 13.04; 27 to 76 years). The most common etiology of polyneuropathy was idiopathic in 50% of cases. Neuropathology reports of sural nerve biopsies showed chronic axonal neuropathy in 9 cases and small-fiber neuropathy in 1 case (Table 1).

Healthy controls included adult patients with no history of neurological disorders, diabetes, or cardiovascular disease. Exclusion criteria for the control group correlated those of the polyneuropathy group, as all patients with peripheral nerve injuries in the last 12 months, and active malignancies or cytotoxic treatments were excluded from the study. Healthy sural nerve tissue was obtained from excess tissue during autograft reconstruction in non-acute peripheral nerve lesions. Sural nerve tissue of 5 female patients were included in the control group. The mean age was 38.4 years (SD 13.4; 28 to 53).

### Nerve biopsies and tissue processing

Nerve biopsies harvested from polyneuropathy patients and controls were processed using standard aseptic technique. The samples were divided in 2 parts, which were approximately 5–10 mm long, depending on the availability of excess nerve tissue. The first part, which was used for immunofluorescent staining, was fixed in 4% formaldehyde for 24 hours. The nerve biopsies were oriented longitudinally and embedded in paraffin blocks. Sections of 8 μm thickness were cut with a microtome (Leica RM2235) and mounted on silane-coated slides.

The remaining part of the nerve biopsies was subsequently fixed in 2.5% glutaraldehyde and processed for histomorphometric analysis of myelinated fibers as previously published [23]. Briefly, the nerve tissue was embedded in epon resin, cut in semithin 1μm sections using an ultramicrotome (Leica Ultracut UCT), mounted on a slide and stained with para-phenylenediamine (Merck).

### Immunofluorescent staining of nerve biopsies

**Fig 1:**
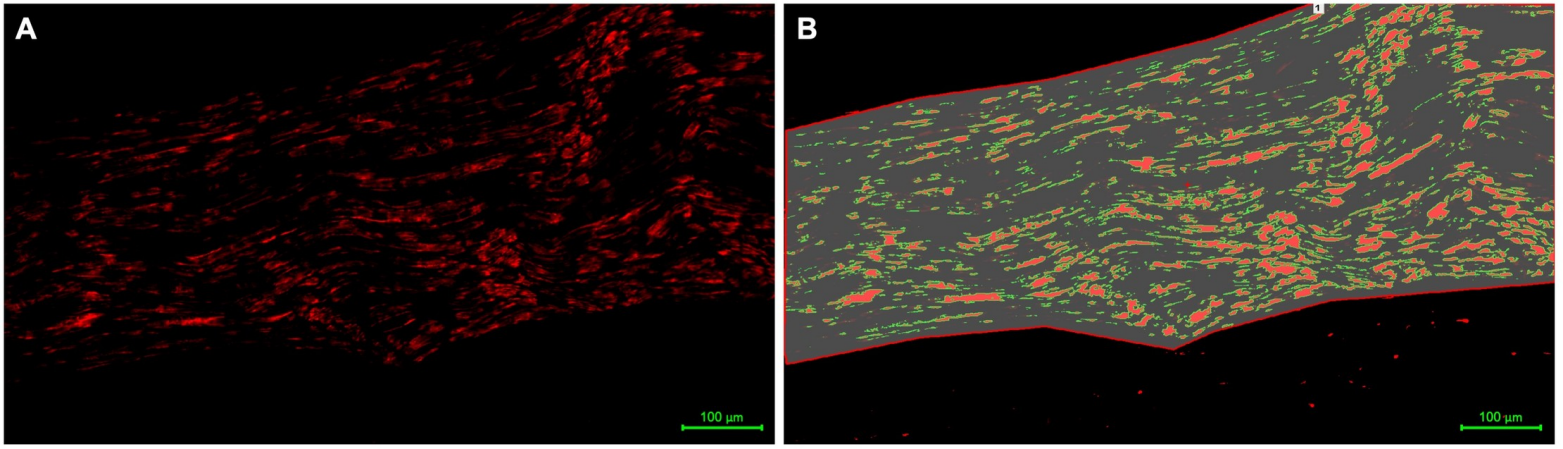
Analysis of immunofluorescent staining. Longitudinal sural nerve section at 20x magnification (S100 antibody; DAKO Z0311; 1:200). NIS Elements AR–Measurement Software was used to measure the intensity of immunofluorescent staining in nerve sections (A). The area fraction of immunofluorescent signals was calculated in the selected area of the image (B). Scale bar: 100 μm.

Nerve sections were deparaffinized, rinsed with phosphate-buffered saline (PBS) and washed with 1% bovine serum albumin (BSA) and 0.2% TritonX-100 in PBS (Sigma). Nonspecific staining was blocked using PBST with 1% BSA and 7% goat serum (Sigma). Primary antibodies were applied and incubated at 4°C overnight. Primary antibodies used in this study were S100 (rabbit polyclonal; DAKO Z0311; 1:200), myelin basic protein (Santa Cruz sz-271524; 1:100), neurofilament-H (Thermo Fisher PA1-10002; 1:300), and anti-p75 neurotrophin receptor (Cell Signaling #8238; 1:500). The slides were washed in PBST and secondary antibodies were applied for 60 minutes at room temperature. The following secondary antibodies were used: goat anti-rabbit Alexa Fluor 594 (Invitrogen; 1:300) for S100 and p75, goat anti-mouse Alexa Fluor 488 (Invitrogen; 1:100) for myelin basic protein, and goat anti-chicken DyLight 650 (Thermo Fisher SA5-10073; 1:300) for neurofilament-H. After using DAPI (Thermo Fisher) for nuclear counterstaining the slides were washed once again with PBS and mounted with a coverslip. Staining intensity was analyzed using NIS Elements AR-Measurement Software, which measured the area fraction of the fluorescent signal in the selected area (Fig 1). Longitudinal nerve sections were analyzed and the area fraction was determined for each individual antibody staining (example shown in Fig 1: S100 staining (rabbit polyclonal; DAKO Z0311; 1:200). The area of interest was selected manually by the investigator.

### Histomorphometric analysis of myelinated nerve fibers

Myelinated axons and the architecture of nerve cross-sections were analyzed at 40x magnification using the NIS software (Nikon; microscope: Nikon Eclipse NI-I; Camera: Nikon DS-Ri2). Fascicle counts, the over-all number of myelinated axons, their density per mm2 and the g-ratio (the ratio of the inner axonal diameter and the outer diameter of the myelin sheath) were assessed manually, or semi-automated depending on the size and homogeneity of the sample.

### Statistical methods

SPSS version 24.0 (IBM SPSS Statistics©) and Prism version 9 (GraphPad Software©) were used for the statistical analysis. Descriptive statistics were performed for all parameters and were reported as mean, standard deviation and range for metric parameters. Student t-tests and one-way ANOVA were performed. The two-sided alpha was set at 5% for all statistical tests.

## Results

### Schwann cells and myelinated axons in nerve biopsies of polyneuropathy patients compared to controls

**Fig 2:**
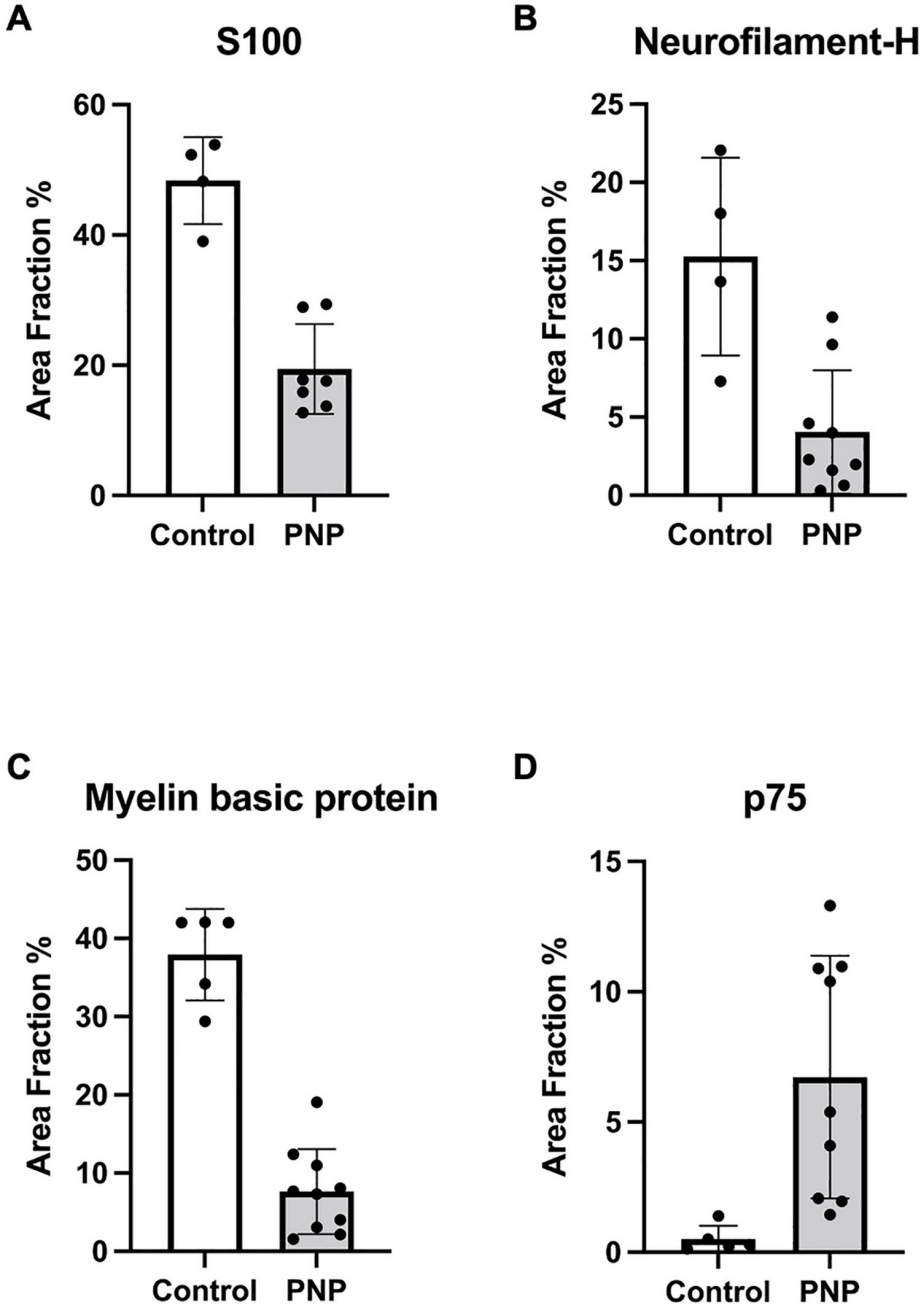
Immunohistochemistry of nerve sections. Area fraction of immunosignals in nerve biopsies obtained from PNP patients compared to controls. S100 positive Schwann cells (A) were significantly reduced in PNP. Neurofilament-H (B) was highly significantly lower in the PNP group. There was reduced immunoreactivity to the myelination parameter myelin basic protein in PNP patients (C), while immature p75-positive Schwann cells were increased (D). *PNP*: *Polyneuropathy*.

In the polyneuropathy group, staining of S100-positive Schwann cells (19.4% ± 6.9, range 12.7 to 29.4) was significantly reduced compared to controls (48.4% ± 6.7, range 39 to 53.9), with a *p*-value of <0.0001 (Fig 2A). The neurofilament-H staining in nerve biopsies of polyneuropathy patients was significantly lower than in controls (4.04% ± 3.9, range 0.3 to 11.38, and 15.25% ± 6.3, range 7.29 to 22.05, respectively) indicating the reduction of nerve fibers in the samples (*p* = 0.0022; Fig 2B). The myelination parameter myelin basic protein was drastically reduced from 37.93% ± 5.85 (range: 29.39 to 42.08) in controls to 7.6% ± 5.4 (range: 1.57 to 19.05) in polyneuropathy patients (*p =* <0.001; Fig 2C). Non-myelinating p75-immunoreactive Schwann cells were identified in polyneuropathy patients (6.7% ± 4.7, range: 1.5 to 13.3), which were significantly increased compared to controls (0.5% ± 0.51, range: 0.13 to 1.39; *p* = < 0.0127; Fig 2D).

**Fig 3:**
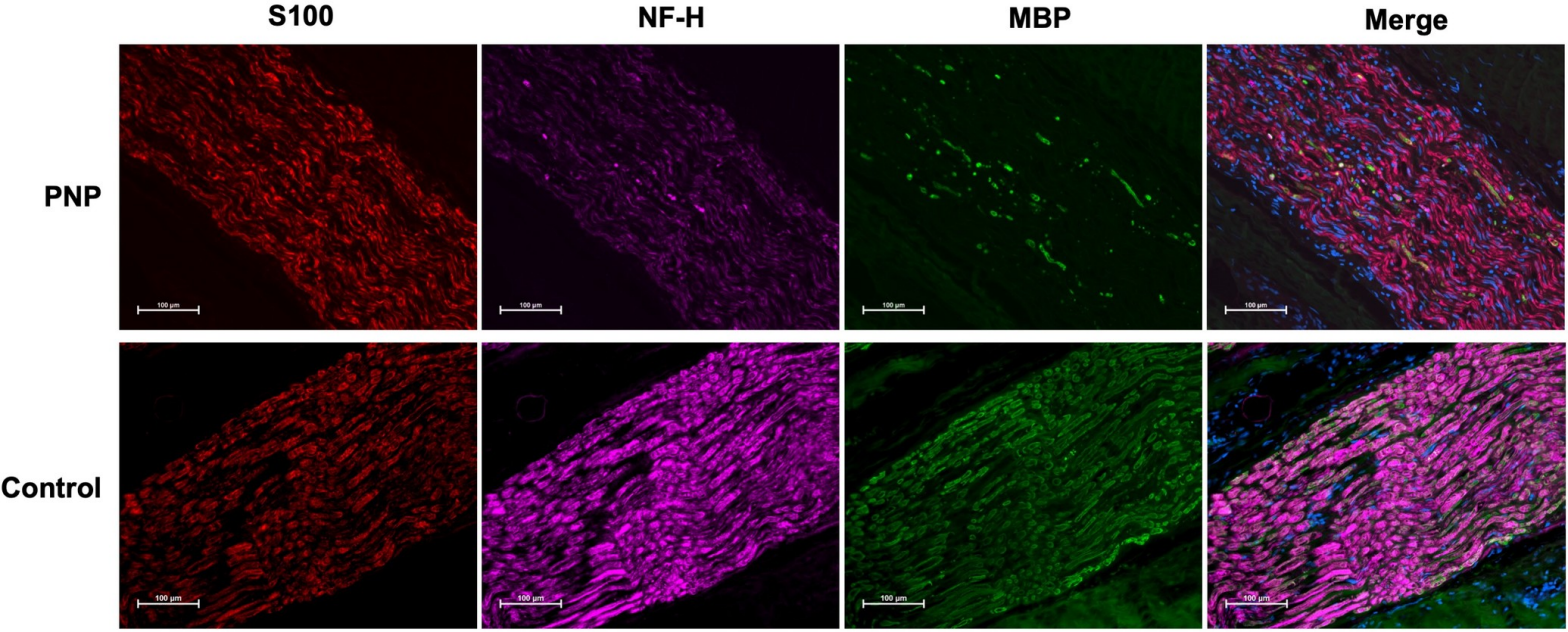
Immunofluorescent staining. S100 positive Schwann cells (red), neurofilament-H (NF-H; purple) and myelin basic protein (MBP; green) are reduced in polyneuropathy (PNP). In the merged panel, cell bodies are counter-stained with DAPI (blue). Imaged at 20x magnification under equal exposure times; scale bar: 100 μm.

**Fig 4:**
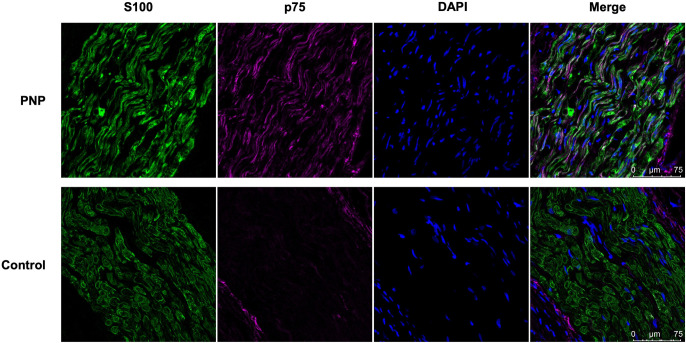
p75-immunoreactive Schwann cells in polyneuropathy. S100 positive Schwann cells (green) and p75-immunoreactive Schwann cells (purple) were stained in sural nerve biopsies. Cell nuclei were stained with DAPI (blue). Imaged at 63x magnification under equal exposure times; scale bar: 75 μm. *PNP*: *polyneuropathy*.

The qualitative images of immunofluorescent staining (Figs 3 and 4) demonstrate the distribution of fluorescent signals in the nerve sections. The reduced signal of S100, neurofilament-H and myelin basic protein in nerve sections of a polyneuropathy patient compared to a control is shown in Fig 3.

The increase of p75-immunoreactive Schwann cells in polyneuropathy compared to a control sural nerve section is demonstrated in Fig 4.

### Histomorphometric analysis of myelinated axons in polyneuropathy compared to controls

**Fig 5:**
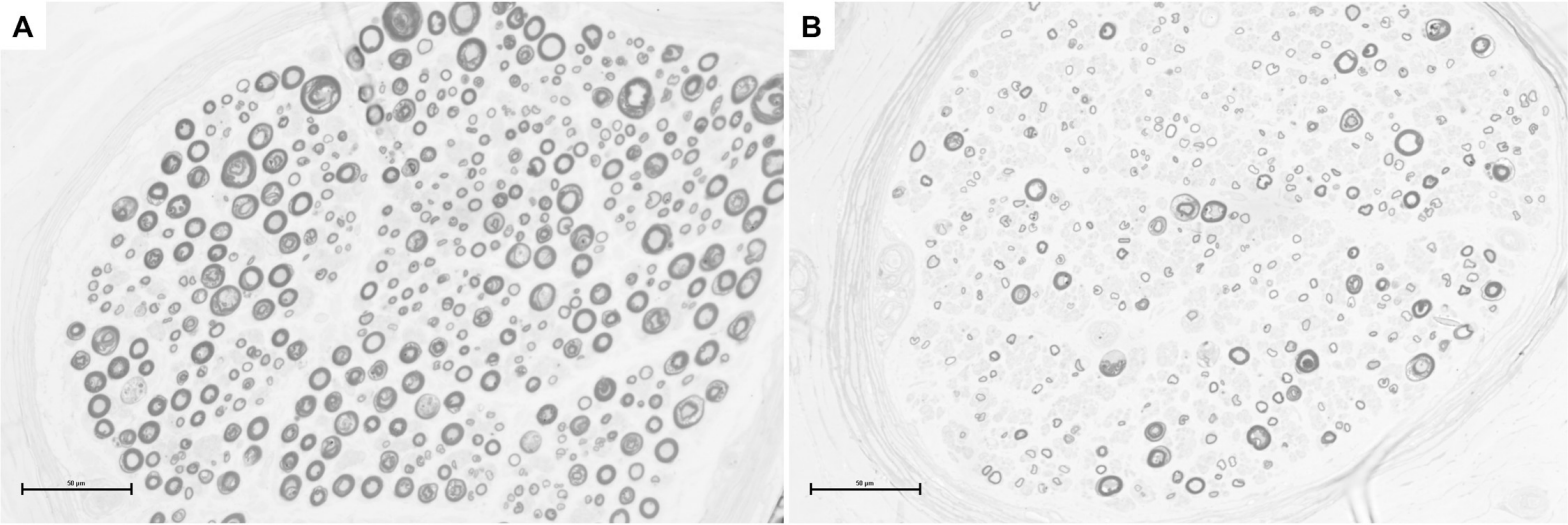
Nerve cross-sections for histomorphometric analysis of myelinated axons. A: Control sural nerve biopsy at 40x magnification. B: Sural nerve biopsy of a male, 68-year old patient presenting with paraprotein-associated chronic, axonal polyneuropathy (at 40x magnification). The number of myelinated axons and their density is significantly reduced. Scale bar represents 50μm.

In cross-sections of nerve biopsies, histomorphometric analysis of the over-all nerve architecture and the myelinated nerve fibers was performed (Fig 5). Sural nerve biopsies of polyneuropathy patients (n = 10) and controls (n = 5) revealed a significant reduction of myelinated nerve fibers with mean axonal counts of 1373.3 ± 1464.6 in the polyneuropathy group compared to 4899 ± 2068 in the control group (*p* = 0.0140). The mean axon density was 2108.3 ± 1635 per mm^2^ in the polyneuropathy group and 9824.2 ± 5294.7 per mm^2^ in controls (*p* = 0.001). The mean g-ratio was significantly lower at 2.7 ± 0.6 in biopsies of polyneuropathy patients compared to 4.248 ± 1.68 in controls (*p* = 0.0360). The mean fascicle counts were not significantly altered in the polyneuropathy group (6.9 ± 2.9) in comparison to controls (4 ± 2.16; *p* = n.s.).

## Discussion

In this study, Schwann cell characteristics and function were assessed in polyneuropathy patients, the majority presenting with axonal polyneuropathy, and compared to neurologically healthy controls. This is the first clinical study reporting human Schwann cell alterations in polyneuropathy, which included a control group. Healthy nerve tissue was obtained from patients who underwent reconstructive surgeries that included selective denervations, like free muscle transplants for soft tissue reconstruction where nerve tissue, which was subsequently processed for this study, would otherwise have been discarded. The stringent inclusion criteria for the control group (no history of cardiovascular disease, diabetes, neurological disease, active malignancy or recent peripheral nerve injury) were chosen to avoid possible bias, as Schwann cells are affected by systemic disease and trauma [18]. Experimental studies showed that peripheral nerve injury leads to upregulation of regeneration associated genes, surface receptors and serum markers [24, 25]. These changes are time-dependent, therefore patients suffering from peripheral nerve trauma within the previous 12 months were excluded. Polyneuropathy patients included in this study underwent diagnostic sural nerve biopsies [1, 22, 26]. Neuropathological ultrastructural analysis was performed as part of the diagnostic algorithm of peripheral neuropathy [27, 28].

### Schwann cell dysfunction in polyneuropathy

The importance of Schwann cell dysfunction in the development and progression of polyneuropathy has been established in experimental models [13]. Early reports of Schwann cell changes associated with polyneuropathy were based on human sural nerve biopsies [20]. In 1992, phenotypic and functional changes in Schwann cells of diabetic patients, the most common subtype in western countries, were reported [29]. These initial studies were mainly descriptive and helped to shape future research which ultimately established Schwannopathy as an independent pathophysiological factor of polyneuropathy [30]. Schwann cell dysfunction in diabetic polyneuropathy is caused by hypoxemia, hyperglycemia and increased oxidative stress, leading to deteriorated myelination, impairment of paranodal barrier function and decreased neurotrophic support [31]. Experimental studies described Schwann cell apoptosis in polyneuropathy models [16]. Reactive, degenerative, and proliferative changes in Schwann cell ultrastructure have been described in affected patients [29, 30, 32]. The current study demonstrated that both significant Schwann cell dysfunction and reduced Schwann cell numbers are found in polyneuropathy patients compared to controls. Immunofluorescent staining showed that S100 positive Schwann cells were reduced by approximately 25% compared to controls. The reduction of myelination as measured by myelin basic protein staining and histomorphometric analysis of nerve cross-sections was even more apparent, indicating significant Schwann cell dysfunction in polyneuropathy patients.

### Demyelination and non-myelinating Schwann cell phenotypes in polyneuropathy

Recent studies described the crucial role of Schwann cells in the pathogenesis and disease progression of polyneuropathy as a key factor next to axonal and microvascular damage [13]. Polyneuropathy is associated with morphological changes of the myelin sheath and progressive demyelination. Reduction of myelination has been measured by decreased expression of myelin basic protein [21, 33]. Our results showed a highly significant reduction of myelin basic protein in polyneuropathy patients compared to controls. Immunofluorescent staining of myelin basic protein in polyneuropathy patients reached only approximately 20% of levels measured in the control group. The g-ratio, a myelination parameter assessed in the histomorphometric analysis of nerve cross-sections, was also significantly reduced in polyneuropathy patients, which concurred with the immunostaining results of the current study.

The “demyelinating Schwann cell” was described as an integral factor in distal polyneuropathies.[34] Alterations in Schwann cell phenotypes occur physiologically during development and peripheral nerve regeneration [35]. In polyneuropathy, Schwann cells undergo phenotypic changes similar to those seen after peripheral nerve injury and chronic denervation, where Schwann cells are deprived of axonal interaction and neurotrophic support [34, 35]. Non-myelinating Schwann cells express several surface markers indicating the transition into a dedifferentiated phenotype [36]. The low-affinity receptor p75^NTR^ of nerve growth factor has been applied as a marker of non-myelinating Schwann cells. Increased levels of the p75 receptor were found in multiple experimental models of polyneuropathy [13, 17, 18, 21, 37]. The current study demonstrated that Schwann cells expressing p75 receptors are significantly increased in polyneuropathy patients, while almost no p75-immunoreactive cells were found in healthy controls.

The phenotypic alterations of Schwann cells found in polyneuropathy patients similar to those seen after peripheral nerve injury emphasize the role of pathological remyelination patterns and impaired nerve regeneration in disease progression of polyneuropathy [11, 13, 34, 38].

### Interactions of Schwann cells and axons in polyneuropathy

The interaction of Schwann cells and peripheral axons are essential not only for myelination but also for neurotrophic support and regeneration after injury and degenerative diseases [14, 35]. *In vitro* studies showed that Schwann cell proliferation and migration, as well as axonal outgrowth from dorsal root ganglia neurons are reduced in hyperglycemic conditions, which is thought to be an essential mechanism in the development of injury-related diabetic neuropathy [18]. Distal axonal loss and impaired regeneration were found in diabetic polyneuropathy [15]. The longest peripheral axons were most affected by loss of trophic support, metabolic stress, and diminished capacity to recover from external injury [14, 37]. Premature apoptosis of dorsal root ganglion neurons and axonal loss were previously described in polyneuropathy [9, 14, 39]. The findings of the current study concur with earlier experimental results as significant reductions of myelinated axon counts and axonal density were found in the polyneuropathy group. Sural nerve biopsies of polyneuropathy patients displayed an approximately fourfold reduction of both myelinated axon numbers and axonal density compared to healthy controls.

### Study limitations and future perspectives

As nerve biopsies in neurologically healthy patients are usually not possible and selective denervations during reconstructive surgeries are a rare exception to obtain healthy nerve tissue, it was not possible to include gender- and age-matched controls for this study. The findings of the present study provide novel quantitative data on human Schwann cell function, myelination and axonopathy in polyneuropathy. Future studies are required to address possible gender-specific differences in Schwann cell dysfunction and axonal alterations, as the control group in this study included all female patients, while a majority of male patients was included in the polyneuropathy group.

The limited patient numbers included in this study did not allow for subgroup analysis regarding the duration and etiology of the polyneuropathy, both factors should be analyzed in larger study populations.

To date, there is limited clinical evidence on peripheral nerve regeneration in polyneuropathy patients. Future studies ought to explore the effect of polyneuropathy on functional outcomes after peripheral nerve surgery. Few experimental studies explored regeneration after nerve graft reconstruction in polyneuropathy [37], however future clinical studies are needed to further explore the role of aberrant or impaired nerve regeneration and the interaction of Schwann cells and peripheral axons in polyneuropathy.

## Conclusions

In this study, Schwann cell dysfunction and phenotypic alterations, as well as myelination and over-all nerve architecture of nerve samples of polyneuropathy patients were assessed and compared to neurologically healthy controls, thereby providing quantitative data on demyelination, non-myelinating Schwann cell phenotypes and axonal loss in polyneuropathy.

## Abbreviations

BSA: bovine serum albumin
CI: confidence interval
CIDP: chronic inflammatory demyelinating polyradiculoneuropathy
MBP: myelin basic protein
NF-H: neurofilament-H
PBS: phosphate-buffered saline
PNP: polyneuropathy
SD: standard deviation
